# Why Do Consumers Make Green Purchase Decisions? Insights from a Systematic Review

**DOI:** 10.3390/ijerph17186607

**Published:** 2020-09-11

**Authors:** Xiaoyun Zhang, Feng Dong

**Affiliations:** School of Economics and Management, China University of Mining and Technology, Xuzhou 221116, China; ts18070012a31@cumt.edu.cn

**Keywords:** green purchase, consumer theory, determinants of green purchase, literature review

## Abstract

In order to achieve sustainable development to protect the environment and society, an increasing number of scholars have conducted in-depth research on green marketing and green purchases. Although great achievements have been made in this field, there still is room for further progress. This study reviews 97 papers providing empirical research on green purchase behavior from 2015 to 2020. First, we review the widely used consumer theory and its extended application in recent years. Second, we divide the influencing factors of green purchase behavior into the following three categories and discuss them in detail: individual factors, product attributes and marketing, and social factors. Finally, we put forward the following possible directions for future research. (1) The authors can consider adjustment to the survey objects to weaken the subjectivity of the data. (2) Longitudinal research can be used to assess the impact of education and policies with a lagging effect on consumers. (3) The authors can broaden the research direction towards a cross-cultural background. (4) The behavior of various green products (such as recyclable tires, recycled glass containers, recycled paper) could be explored to enrich the research content. (5) It will be beneficial to combine a variety of consumer theories to explore the green purchase behavior of consumers and break through the existing linear hypothesis path to explore new research methods.

## 1. Introduction

With increasing threats to the environment, an increasing number of people have started to pay attention to sustainable development to protect the environment and society. The concepts of green purchase and green marketing have gradually become popular. Green purchase refers to the green product purchase activities conducted by consumers to save resources and protect the environment [1]. Green marketing refers to the marketing activities (including price, plan, process, production, promotion, and personnel) designed by enterprises for all consumers. The purpose of these activities is to minimize the environmental impact of the company’s products and service [2]. Many companies have changed their production strategies to actively respond to environmental problems and changes in consumers’ environmental attitudes. They abandoned products that are relatively polluting to the environment or harmful to human health and turned to environmental protection products. Although manufacturers have produced environmentally friendly products, many consumers are not interested in their products due to their consumption values, resistance to new technologies, or their resistance to the premium prices charged for these new products, which leads to sales obstacles for these products [3,4,5]. Therefore, it is of great practical significance to understand the influencing factors of consumers’ green product purchase behavior for green marketing.

The theoretical research on green marketing has a long history involving psychology, economics, philosophy, management, marketing, and other disciplines. Most consumer theories that explore the impact of individual factors on green purchase behavior believe that environmental knowledge and values have an impact on green purchase behavior by influencing consumers’ environmental attitudes [6,7,8,9]. There are also some consumer theories to explore the impact of individual factors and external factors on green purchase behavior. Some of them think that individual and external factors both have a direct influence on green purchase behavior or that the latter indirectly affects consumers’ purchase behavior through influencing the former [10,11,12], while the others hold the opinion that external factors play a moderating role between individual factors and purchase behavior [13,14]. Groening et al. [2] systematically reviewed more than 20 consumer theories according to six dimensions: environmental values and knowledge, beliefs, attitude, intentions, social information, and motivation. Their main contribution is to build a framework from many theories, which indicates the flow between the theory groupings. Future researchers can get inspiration from Groening’s [2] framework of green marketing and green consumerism theoretical relationships when exploring the consumer behavior at a certain stage.

In addition to taking the consumer theory as the research framework, many researchers have studied consumers’ green purchase behavior from a certain perspective (such as consumer’s emotion, perceived consumer effectiveness, connected to nature) or put consumers in a specific situation (such as background of environmental co-governance, social interaction) [15,16,17,18]. As for the influencing factors of consumers’ green purchase behavior, some scholars have carried out a systematic review from different research perspectives. Bangsa and Schlegelmilch [19] mainly reviewed and summarized the impact of green product attributes on consumers’ purchase decisions by dividing the product attributes into social sustainability attributes and environmental sustainability attributes. Yatish and Zillur [20] systematically reviewed 53 research papers from 2000 to 2014 and divided the influencing factors of green product purchase behavior into individual factors and situational factors, among which the situational factors were product attributes, packaging, and others. Eighty empirical papers which range from 2011 to 2017 on the green consumption of consumers were analyzed by Liobikienė and Bernatoniene [21]. They classified the influencing factors of green purchase behavior into internal factors, social factors, and external factors and discussed the influencing factors of green purchase behavior according to the classification of green products. When reviewing the influencing factors of green purchasing behavior, the above-mentioned scholars classified them into individual factors and external factors, of which external factors are divided into product factors and social factors, which provide an overall conceptual framework for related research.

Referring to the previous literature review, we mainly made the following contributions. First, we used Scopus as the main database to conduct a detailed review and systematic analysis of papers from 2015 to 2020, which extended the research time range. Second, although some scholars have reviewed the green consumer theory at the consumer level, their comments are an overall review of all the consumption theories. They have not studied the revision and expansion of these models, and have not pointed out which models have the highest applicability or the most extensive application range. Nor have they considered the mixed use of models. We have tried to analyze which are the high-frequency consumer theories and the expansion of these, and explored the mixed use of some theories. Third, we have classified the influencing factors of consumers’ green purchase behavior in the context of fragmentation, and divided them into individual factors, product attributes and marketing, and social factors in order to inform future research.

The remainder of the paper is arranged as follows: The research methodology is described in Section 2. Section 3 describes the findings. The discussion of this paper is presented in Section 4. Finally, we summarize our research, limits, and future development direction in Section 5.

## 2. Research Methodology

### 2.1. Selection Process

Green product, green purchase, green buying, sustainable products, sustainable consumption, sustainable purchase, and willingness to pay were used as the keyword structure to search the relevant papers published in 2015 to 2020, with Scopus being the main database. The selected papers were filtered by the following three conditions. (1) The papers were published between 2015 and 2020. (2) They must be empirical papers to ensure the objectivity of the research results. (3) The main content of the research was to explore the green purchase behavior of consumers, which includes not only the consumer theory but also the influencing factors. Finally, 97 papers were selected as the main research scope.

### 2.2. Content Analysis

Before discussing the literature, we developed detailed descriptive statistics on the 97 selected papers to further analyze the research content of each article. The content of the descriptive statistics is as follows. (1) The papers were classified according to the time period, journal, survey region, and the category of green products. (2) The consumer theories with high frequency of use were statistically analyzed. (3) The consumer green purchase factors were divided into three categories: individual factors, product factors and marketing, and social factors. There may be repetition in the classification because a paper may involve many influencing factors or green consumer theories when we classify and analyze the content of the papers.

### 2.3. Material Evaluation

**Figure 1:**
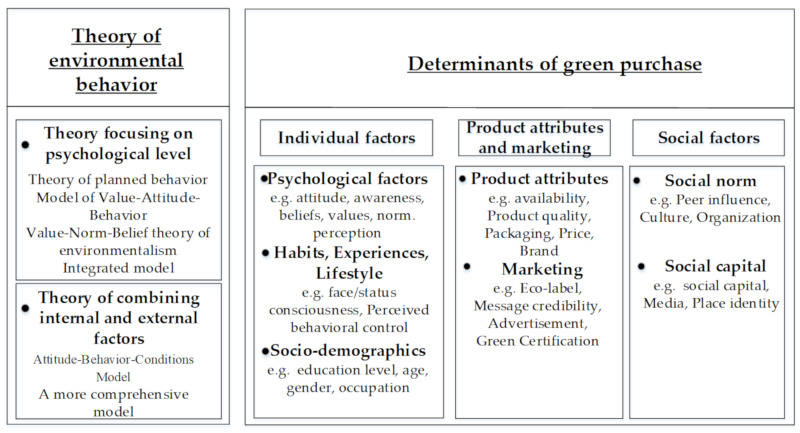
Framework of material analysis.

The material analysis was divided into two aspects. In the first part, on the basis of the other related literature, we summarized the widely used consumer theories: Theory of Planned Behavior (TPB), Value–Attitude–Behavior Model (VAB), Value–Norm–Belief Theory of Environmentalism (VBN), the integrated model, the Attitude–Behavior–Conditions Model (ABC) and more comprehensive models. In the second part, the specific green purchase behavior factors were classified and summarized into three indicators: individual factors, product factors and marketing, and social factors. The individual factors included psychological factors, lifestyle, experience, and habits. Social factors were subdivided into social capital and social norm. According to the research results of the empirical papers, we discussed the influence of each variable on green purchase behavior. The material analysis framework of this paper is shown in Figure 1.

## 3. Findings

### 3.1. Distribution Across the Time Period, Journal and Region

The 97 papers selected in this review are distributed from 2015 to 2020. They were published in various journals and involved various research types, such as energy, production, food, transportation, clothing, and management (see Appendix A), which reflects the interdisciplinary nature and complexity of green purchase behavior. The review covers the first half of 2020, in which there are 15 empirical studies on green purchase behavior in Scopus. This shows that the research on green purchase behavior still has important practical significance.

Culture is of great significance to an individual’s value orientation and should not be ignored in the study of consumer behavior. Thirteen papers were completed in a cross-cultural context, of which six were cross-country studies in Europe, five were in the United States, and the others were cross-country studies between China and Germany and Australia and Lithuania (see Appendix B). There are only four cross-cultural studies between developing countries and developed countries, and these are comparisons between America and India (two papers), America and China, China and Germany [8,22,23,24]. There are significant differences in culture among different countries, especially between the Eastern and Western countries. Obviously, there is not enough research on this aspect. As for the research on the green purchase behavior of consumers in a single country, most of it is on China, with a total of 29 papers, followed by India, with a total of 14 articles. Among the European countries, there are six papers on German and Italian consumers. The other countries with more research are Malaysia, South Korea, and Brazil. From the perspective of the distribution of the research region, developing countries are paying increasing attention to environmental problems, but the research in a cross-cultural context needs to be supplemented.

### 3.2. Content of Papers

#### 3.2.1. Research Based on Green Product Category

The 97 papers mentioned above studied the purchase behavior of green housing, furniture, household appliances, vehicles, food, clothing, and other products. Among the 97 papers, 57 have no clear typology with regard to the green products, whose dependent variables are the collection of purchase behaviors or the purchase intentions of green products such as energy-saving products without excessive packaging, products without much environmental pollution, and recyclable products. The remaining 40 papers specifically studied the purchase behavior of a certain type of green product. Clothing, food, housing, and transportation are the four elements of human life, and almost all human activities are carried out around them, which have produced a large amount of carbon dioxide [25]. Therefore, it is of great significance for sustainable development to improve the green degree inherent in clothing, food, housing, and transportation.

**Figure 2:**
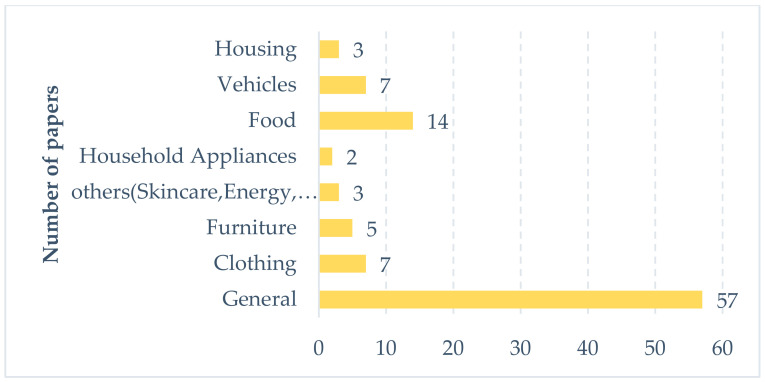
Distribution of papers across product category.

First, we made a quantitative analysis of 97 literatures mentioned above according to the product categories. As shown in Figure 2, the research on food is the most abundant, with a total of 14 articles. The number of papers on clothing, vehicles, housing, furniture, and appliances is 7, 7, 3, 5, and 2, respectively. In addition, there are also three studies on skincare, bioplastic products, and green energy. Then, we made a systematic statistical analysis on the research region, consumer theory, and key variables of the abovementioned 40 papers according to the categories of food, clothing, vehicles, housing, furniture, appliance, and other categories (see Table 1).

With regard to food, quality is one of the most concerning factors for consumers. More and more consumers are increasing their consumption of organic products for the sake of health [26]. However, consumers usually choose their green products or a certain brand due to their previous purchase experience or low cost [5,27]. Vehicles are durable goods for ordinary families, so their purchase decisions often need to be carefully considered. The cost-performance ratio is usually the key factor affecting consumers’ purchase of vehicles [14]. There are also some consumers who choose to purchase environmentally friendly vehicles to show their identity or to obtain a good social image [28]. As for clothing, as one of the most direct representatives of personality, its aesthetics has a greater impact on product selection, but its practicability has a relatively small impact on decision-making [3,29], and even product durability preference has a negative effect on consumers’ purchase behavior of sustainable clothing [30]. Housing is an important fixed asset for families, so the behavior in respect to purchase intention is one of caution. The purchase choice is usually made according to the living needs of the family members [13]. Furniture is generally a household durable product that is usually retained for several years or even decades, so the properties and quality of it are important considerations in the purchase decision. To fully mobilize the enthusiasm of consumers, it is necessary to strengthen the publicity of such products in respect of their health attributes and harmlessness when focusing on the environmental protection properties of green furniture [7,9,31].

As shown in Figure 2, compared with the research of clothing, food, housing and transportation, the research on other categories of products is obviously less. In the future, scholars could carry out a lot of research on purchase behavior of other products (such as recyclable tires, recycled glass containers, recycled paper), so as to promote the sales of such environmentally friendly products and promote sustainable development.

#### 3.2.2. Widely Used Consumer Theory

The research on environmentally friendly behavior involves environmental psychology, environmental pedagogy, environmental sociology and other specialized fields. The theoretical framework of some environmental behavior has strong applicability in the study of green purchase behavior, because green purchase is a typical private environmental behavior. Most scholars have discussed consumer theory from the aspects of psychology and the combination of internal and external factors. Theory of Reasoned Behavior, Theory of Planned Behavior, Norm Activation Model, and Models of Value–Attitude–Behavior and Value–Belief–Norm focus on the study of environmental behavior from the psychological aspect, while the Attitude–Behavior–Conditions Model explores green purchase behavior from both social and psychological aspects, so as to better demonstrate the influence of social situation and social structure on purchase behavior (see Table 2). Researchers directly added new variables or adjusted the function path of the original theory to improve the contribution of the theoretical mechanism to the interpretation of practical problems. While we are analyzing the expansion and application of the single consumer theory, we also further explore the use of the mixed model, which provides a new idea for the future research direction.

#### 3.2.3. Determinants of Green Purchase

There are many factors influencing green purchase behavior, and these are divided into three dimensions: individual factors, product attributes and marketing, and social influence (see Table 3).

Among them, individual factors are divided into three aspects: psychological factors, habits and lifestyle, and socio-demographics. Psychological factors are mainly composed of attitude, environmental consciousness, and beliefs and values. Habits and lifestyle are subdivided into face/status consciousness, health status, healthy lifestyle, and so on. Socio-demographics are mainly reflected in education level, age, gender, occupation, and family structure. Product attributes mainly involve product quality, price, perceived risks, and trust, while marketing is composed of eco-label, advertisement, and green word-to-mouth. As for social influence, it is divided into social norm and social capital. Social norms are measured from the perspective of peer, culture, and organization. Social capital mainly measures social norms from social media and place identity.

First, as for the personal factors of consumers, on the one hand, the differences in gender, age, education, and work make consumers have different levels of demand for green products. On the other hand, the psychological variables have a profound and lasting guiding effect on consumer behavior, which leads to significant differences in consumers’ purchase intention or behavior due to their different attitudes and expectations in respect of environmentally friendly products. Second, the attributes of green products are also key factors that consumers should consider when purchasing goods. Products should not only meet the practical and price needs of consumers but should also provide the environmental benefits expected by consumers. The marketing strategies also play an important role in guiding consumers’ purchase intention. The biggest difference between green products and traditional products lies in their green attributes. Businesses should not only highlight the sustainable impact of green products on the environment and on social development but they should also pay attention to the authenticity of the information in their publicity to avoid false publicity such as “greenwashing”, which would reduce consumers’ trust in green products. Our specific analysis is briefly summarized in Section 4.2.1 and Section 4.2.2.

As social people, consumers have been imperceptibly influenced by the social environment, such as the government’s policies, media reports, and the various environmental protection organizations. There are great cultural differences between Eastern and Western countries. Chinese people pay attention to harmony and think that collective interests are higher than personal interests. However, American people regard personal interests as the most important goal. Great differences in product choices have been inevitable due to the different values pursued by the two countries’ people. Therefore, it is of great significance to examine green purchase behavior under different cultural backgrounds, which has important practical significance. A more detailed analysis is summarized in Section 4.2.3.

## 4. Discussion

### 4.1. Consumer Theory

For a long time, the theories represented by Planned Behavior Theory, Value–Attitude–Behavior and others mainly explore the influence of psychological factors on consumers’ green purchase behavior. Until the mid-1990s, more and more scholars brought external factors into the theoretical model and studied consumers’ green purchase behavior from internal and external factors.

#### 4.1.1. Widely Used Consumer Theory Based on Psychological Factors

The green consumer theories focusing on psychology hold the viewpoint that people’s pro-environmental behavior is mainly determined by psychological factors, which pays great attention to the influence of the perceived behavioral control, attitude, values, moral norms, and other factors on consumers’ purchase intention or behavior regarding green products. Even if sociodemographic factors and other external factors have an impact on behavior, their roles are mainly through the indirect influence of belief, motivation, and other psychological factors [93].

(1) Theory of Planned Behavior

The Theory of Reasoned Action (TRA) asserts that people will think about the consequences of their behavior rationally before making behavior decisions [93]. Attitude indirectly affects behavior through intention, which is influenced by the subjective norm. The TRA was extended by Ajzen [94] who added the perceived behavioral control and proposed the Theory of Planned Behavior. Ajzen believes that people should not only consider the consequences of their behavior but they should also consider whether they have the ability to do this behavior before making behavior decisions.

The effectiveness of the TPB’s interpretation of behavior has been confirmed by a large number of empirical studies, and it has become the main theory used to study green purchase behavior [38,95,96]. Rezai et al. [97] studied 1355 Malaysian consumers’ purchase behavior of green food using TPB, and their empirical result shows that attitude, subjective norms, and perceived behavioral control are the main variables contributing to purchase behavior. Similar results were obtained by Madeline et al. [41] when they predicted consumers’ purchase of green housing. However, because the explanatory power of TPB is limited, more and more psychological variables have been added into it to explore the motivation of green purchase behavior [9,36,37,38,98]. In addition, some psychological factors, such as consumer innovation, environmental concern, and perceived value have been added into TPB as pre-variables or mediating variables to improve the explanatory power of the model, which not only improves the interpretation of the original model but also greatly broadens the research perspective of TPB in the field of green consumption behavior [38,98,99,100].

The authors found that TPB has been the most widely used in recent years. However, there are still limitations in the application of TPB in the study of green consumption behavior. First, TPB assumes that the behavior subjects are rational. However, most consumers generally continue their previous purchase habits when purchasing commodities, except for bulk commodities such as automobiles, residences, and home appliances, and TPB cannot comprehensively explain this consumption behavior. Therefore, when using TPB to study consumer’s purchase behavior, we can add the past purchase experience, purchase habits, and other non-rational factors to explore the influence of irrational factors on the purchase behavior. Second, TPB mainly focuses on the influence of the psychological level on behavior but ignores the effect of external factors. Although scholars have expanded it, it is still difficult to get rid of its research paradigm of a fixed linear attitude–intention–behavior.

(2) Model of Value–Attitude–Behavior

The validity of Value–Attitude–Behavior was verified by Schwarts [101] through their investigation on consumers’ purchase intention for food. Sheth et al. [102] made an in-depth study on the effect of values on consumer behavior and proposed the Theory of Consumption Value, where functional, social, emotional, conditional, and epistemic values were used to answer the question of why consumers choose to buy (use or not use) a particular product. The applicability of this theory in green purchase behavior has been widely verified [73,103,104,105].

VAB, an effective consumption forecasting model, has been widely used as a theoretical framework in the study of green purchase behavior [11,46,49]. Some scholars have directly added variables, such as green product information, environmental performance and confidence, price sensitivity, individual environmental literature, and external motivating factors, into VAB to study their direct impact on green purchase behavior [6,30,45]. Intermediary or moderating variables, such as green product quality and place identity, were also added into the VAB framework, which revised the original path of VAB [6,48]. The development of the VAB model has not only improved the explanatory power and applicability of the model, but it has also facilitated a deeper level of examination on the research problems.

(3) Value–Norm–Belief Theory of Environmentalism

The Norm Activation Model asserts that the awareness of adverse sequences and description of responsibility activate consumers’ personal norm, which directly affects consumers’ pro-environment behavior [101]. Stern [106] later combined the Value Theory and New Environmental Paradigm (NEP) with the NAM mediation model to propose the Value-Belief Theory of Environmentalism (VBN), in which value is subdivided into biospheric, altruistic, and egoistic. Responsible environmental behavior is more likely to be taken by the people who pay close attention to the environment and society and who believe that human activities and the fragile ecology are an inseparable whole (NEP) [107,108].

Pro-environmental behavior was divided into four categories: activism, nonactivist public-sphere behaviors, private-sphere behaviors and behaviors in organizations [106]. The VBN theory has strong applicability, because green purchase is a typical kind of environmental behavior in the private domain [109,110]. In order to improve the interpretation of the VBN model for environmentally friendly behavior in the private domain, scholars have extended or modified it by adding or replacing new influencing factors. For example, two emotional factors, as moderating variables, were added into the direct-action path of AC-PN when Han et al. [51] used VBN as the theoretical framework to study consumers’ environmental behavior. The results showed that the expanded VBN model had a better capacity to predict the final behavior.

VBN is a mature model, and it has been widely used in the study of green purchase behavior. However, the variables involved in the model are all psychological factors. Both VAB and VBN ignore the influence of external environmental factors on behavior/intention. Even though scholars have expanded VBN, there is still much room for improvement in its simple behavior model to explain consumers’ green purchase behavior in the social environment.

(4) Integrated Model

As shown in the above analysis of the various models, each has different emphases. TPB emphasizes that human beings make decisions based on rational thinking. The influence of the value on purchase behavior was concentrated on in VAB, whereas VBN and NAM regard personal norms as the most direct influencing factor on environmental behavior. Various theories have been combined to explain consumer behavior more fully because of the complexity of the psychological factors and the influencing mechanism of personal environmental behavior. The more classic of these is the compound model proposed by Bamberg and Moser [111] via a meta-analysis on the psychological and social determinants of pro-environmental behavior.

**Figure 3:**
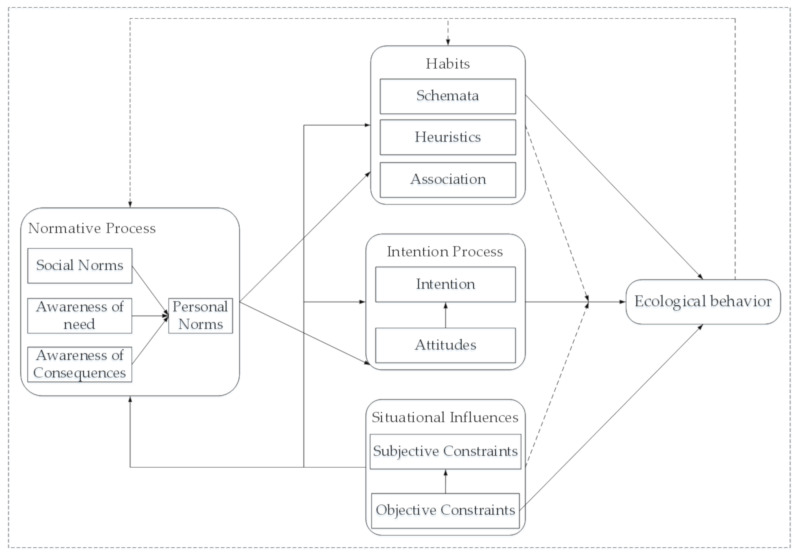
A comprehensive action determination model.

A more comprehensive study on the model, which integrated TPB, NAM, and VBN, as well as adding some other variables (habit and situational influences) was then made by Klöckner and Blöbaum [112], and the Comprehensive Action Determination Model (CADM) was proposed (see Figure 3). Compared with the previous model, CADM has a more perfect structure and involves more variables. Green purchase behavior is one of the typical pro-environmental behaviors in the private field. The CADM is a more comprehensive research paradigm of the psychological mechanism. At present, there are few papers where this model has been used to study green consumption behavior, and the applicability of it needs to be verified. Future researchers can use this model as a reference to propose a higher-explanation model when designing an empirical structure.

#### 4.1.2. Consumer Theory Based on The Combination of Internal and External Factors

With the deepening of the research on environmental behavior, scholars’ theoretical research on environmental behavior has not been limited to the psychological aspects. External environmental factors have gradually been included to study their indirect or regulatory role between the psychological factors and environmental behavior. Some researchers have also studied the direct impact of external situational factors on environmental behavior.

(1) Attitude–Behavior–Conditions Model

Stern and Oskamp [113] believed that responsible environmental behavior is the ultimate goal of education, and they proposed their model of responsible behavior. They held the view that environmentally friendly behavior is the result of the interaction of a series of external factors, including the social structure, social system, and economic mechanism, as well as internal factors, such as specific and general environmental attitudes, beliefs, and behavioral intentions. Guagnano et al. [114] simplified the Model of Responsible Environmental Behavior into the Attitude–Behavior–Conditions Model. Three variables: behavior, attitude, and conditions, are incorporated in the ABC model, in which consumers would adopt purchase behavior only when attitude and conditions are satisfied.

Green purchase behavior, as a kind of pro-environment behavior in the private field, is influenced by psychological factors and external situational factors. The lifestyle, attitude, social norms, area of residence, and other internal and external factors have been combined to study consumers’ purchase behavior in respect of sustainable food, the empirical result of which showed that the above factors had a significant impact on consumers’ green purchase behavior. Attitude and environmental concern had the greatest effect and were followed by lifestyle [53]. In addition, the impact of the product attributes on consumers’ green purchase behavior cannot be ignored, especially the green attributes and brands of products, which have a profound guiding effect on consumers’ product selection [5]. Megavannan et al. [54] divided the factors influencing consumers’ green product purchase behavior into two categories: ecological consistency of green products and challenges to buy green products. Their empirical result indicates that the credibility of the eco-label, identity of the green products, quality, and awareness about green products all have significant impacts on green purchase behavior, but the influence of environmental consciousness is insignificant. Furthermore, some studies have taken into account the influence of consumers’ psychological factors and external factors, such as the media and social interaction on consumers’ purchase of environmentally friendly products, and these studies found that both variables have a significant impact on purchase behavior [55].

The ABC model provides a framework for scholars’ research on green consumption, but it does not specify which external factors and psychological factors have a direct or indirect impact on green consumption behavior or intention and through which path. A large amount of empirical analysis is still needed in the future

(2) A More Comprehensive Research Model

**Figure 4:**
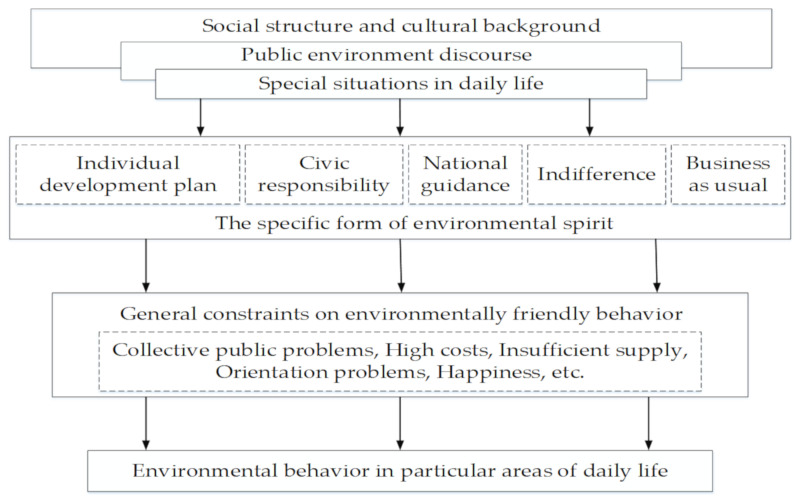
Context model for analyzing environmental consciousness and behavior.

Green purchase behavior, as a kind of private environmental behavior, is mainly affected by psychological factors, but the subtle influence of the social structure, cultural background, and other life situations on consumer consumption values cannot be ignored. Peng [115] proposed a multi-dimensional, selective, and environmental model to explain the relationship between environmental awareness and environmental behavior on the basis of the graphics and related content in Brand’s [116] article (see Figure 4).

Consumption values, being a kind of social culture, are determined by consumers’ environment and education. Taking Chinese consumers as an example, they have been immersed in a Confucian culture for a long time and pursue social harmony, so they are more likely to implement environmental behavior [117]. In addition, social and natural conditions, such as the development structure and topography, also affect consumers’ attitude towards green products. Habich-Sobiegalla et al. [118] analyzed the purchase intention of Chinese residents for electric vehicles from the macro and micro levels. China has a vast territory where there are great differences in the development structure and economic conditions among regions. The residents in the more developed and relatively flat eastern region have higher purchase intentions in respect of electric vehicles than those in the relatively backward and vast western region. Urban residents living in densely populated areas are more willing to buy electric vehicles than those living in rural areas who have different regional and health issues, which indicates that the living environment has an important impact on consumers’ choice of products.

We found that the questionnaire methodology is the main means for scholars to study consumers’ green product purchase behavior. Although questionnaires can be used to collect and quantify consumers’ cognition and attitudes towards the environment and green products, it is difficult to quantify the social structure, production mode, and social differentiation because of their abstract nature. Moreover, questionnaire surveys collect cross-sectional data about consumers, which makes the dynamic influence of education, policy, and other contextual factors with lag effects in the process of consumers’ green product purchases difficult to explain. Therefore, future scholars should explore new research methods.

### 4.2. Determinants of Green Purchase

**Figure 5:**
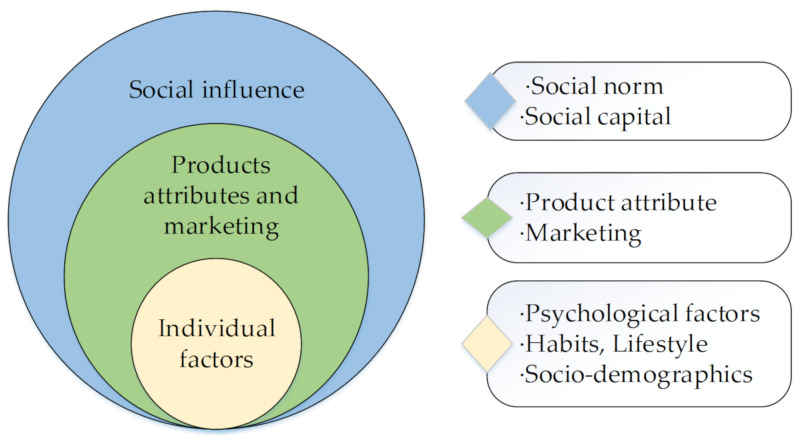
Classification of determinants of green purchase.

Most of the papers reviewed in this study regard green purchase behavior as a rational behavior of consumers to protect the environment at the expense of part of their personal interests. As shown in Figure 5, the research work focuses on the consumer’s psychological factors (e.g., attitude, values, mores, norms), behavior habits and lifestyle (e.g., past purchase behavior, knowledge), as well as socio-demographic influences (e.g., age, gender, education, occupation) on consumers’ green product choices [47,61,119]. Of course, consumers cannot buy products just based on their own likes and dislikes. The product quality, attributes, and marketing strategies of businesses are all factors that consumers should pay attention to when choosing green products. Taking green food as an example, consumers focus on food safety and health [5], but consumers are more concerned about style and personality in terms of clothing [29,30].

However, the formation of consumer values is closely related to their living environment, because we all live in groups, which indicates that individual factors and product attributes cannot fully explain the consumer attitude behavior gap. Therefore, scholars have included more macro factors, such as the social development, production structure, and culture, in their research [80,118]. The authors classify the influencing factors involved in empirical articles into three categories: individual factors, products attributes and marketing, and social influence (Figure 5), and they discuss them in detail.

#### 4.2.1. Influence of Individual Factors

First, socio-demographic variables, which are objective factors, are generally used as the classification variables to compare and analyze consumers’ green purchase behavior or intention. Liobikiene et al. [88] found that compared with men, women are more likely to care about other people’s lives and are more willing to buy green products. However, some studies have shown that gender does not have a direct impact on green purchase, and there was no significant effect between attitude, perceived behavioral control, subjective norm, and green purchases when gender was taken into account as a mediator [14,35,58,118]. According to the results of the study on the purchase intention in respect of electric vehicles, middle-aged people aged 35–49 have the highest purchase intention, while those younger than 19 years old have the lowest [118]. What causes this may be that most middle-aged people are the main source of the family income, so they pay a lot of attention to the purchase cost and quality of products when purchasing goods. Furthermore, electric vehicles are mainly driven by electricity, which can save fuel costs. However, juveniles under the age of 19 have only a vague concept of money and pay more attention to the personality characteristics of products. It is difficult for the performance of electric vehicles to meet their individual needs. In addition, there are significant differences in the purchase intention among different income groups regarding environmentally friendly vehicles. People in the middle-income level have the highest purchase intention for electric vehicles, while rich people do not because they want to show identity or pursue better driving performance through luxury cars [118].

We also found some other interesting phenomena when we combed the literature. In contrast to the finding that age and income have great differences in the purchase decision of electric vehicles, people with different ages and incomes have not exhibited the expected significant differences in respect to purchasing green food [55]. We speculate that there are two reasons for this difference. On the one hand, consumers have different demands for cars with different properties, whereas the perception of environmental protection and health attributes of green food are almost the same. On the other hand, consumers make rational plans and estimations before purchasing different vehicles due to their large price. However, the price of green food is relatively cheap, which indicates that the behavioral cost difference between consumers is so small.

There are also different findings on whether there is a relationship between occupation, family structures, and consumers’ green purchase intention [55]. There is no unified conclusion about the education level and consumers’ purchase behavior towards green products. Some studies show that the higher the education level, the more willing consumers are to buy green products [12,82]. However, Fleith et al. [31] found that there was no relationship between the education level and the social benefits of environmentally sustainable products. We speculate that this phenomenon was caused by the fact that the education level of the subjects surveyed by the authors had met the threshold education level of green consumption, so the effect of education was not significant. Later, scholars should pay attention to the classification of consumers by different ages, incomes, or education levels in their studies of green purchase behavior so as to provide references for businesses to develop efficient and targeted marketing strategies.

Attitude is generally considered to be the most direct factor influencing consumers’ green purchase behavior or intention, and the relationship between attitude and green purchases has always been the most studied topic from the abovementioned consumer behavior theories [45,65]. Attitude is subdivided into attitude toward the environment and attitude to products, and both have significant influences on green purchase behavior [10,120]. Generally speaking, the environmental benefits brought by green products can encourage consumers to buy green products compared with the social benefits brought by green products [6]. However, Xu et al. [9] drew a conclusion contrary to most researchers in the study of consumers’ purchase intention of green furniture: Attitude regarding purchase intention for sustainable furniture is not significant; on the contrary, the positive effect of positive behavioral control on consumers’ purchase intention is the greatest. Sometimes the effect of perceived behavioral control on consumers’ green purchase behavior is stronger than their attitude, because consumers cannot choose products entirely based on their own preferences, such as price, availability, and other factors [26]. In particular, when purchasing green housing, furniture, and appliances, consumers’ consumption power is often more critical than their environmental and product attitudes [9,41,47].

At present, the price of green products in the market is relatively higher than that of traditional products, but they still occupy a place in the market mainly because people attach much more importance to environmental issues, and they are willing to pay a green product premium for the purpose of environmental protection [27,40,46]. In the past, human beings wantonly exploited and wasted resources with the slogan of conquering nature. The rapid development of industry and the increasing volume of domestic garbage have led to serious environmental pollution problems, as well as various diseases and hidden dangers in life. Facing the “revenge” of nature, human beings began to change their thinking and behavior. They began to take the protection of nature as their responsibility so as to live in harmony with nature. Driven by this new mindset, people will have a sense of guilt and shame for their actions that may damage the environment, so they will consciously avoid doing harm to the environment when purchasing products [63,67]. Generally speaking, people with higher ecological literacy are more willing to pay more for environmental protection products [60]. Dong et al. [16] studied the green purchase behavior of consumers from the perspective of the relationship between humans and nature, in which a commitment to nature reflects the human love for nature. Those who love nature think that green products are in line with their values and can achieve the goal of sustainable social development. Therefore, they have a more obvious purchase tendency for green products [67,89].

It is impossible to make a rational estimation before purchasing every product because consumption is one of the most common daily behaviors of individuals. Especially for the consumption of products with low behavioral costs, such as food and clothing, consumers’ motivation in product selection is more driven by previous behavior habits, purchase experiences, or lifestyle [26,27,36]. An individual’s lifestyle and life habits are hard to change in the short term, and they affect consumers’ demand level, brand preference, consumption location, and shopping style. For example, people who never smoke have higher green consumption intentions than those who smoke. People who pay attention to health have a strong tendency towards organic products, and people who read books and newspapers are also more likely to accept and buy green products [65,121].

Purchase behavior of product in daily life is highly repetitive. Consumers usually make repeat purchases of products with which they have had a good experience over time, and green products are no exception. It has been found that compared with the attitude and personal norms of consumers, the past purchase experience of organic food has the greatest impact on the purchase intention of organic food in the future [27]. Khare and Sadachar [68] also drew a similar conclusion when studying the purchase behavior of sustainable clothing: the correlation between past environmental behavior and purchase behavior is higher than that between peer influence, apparel knowledge, and green apparel buying behavior. In addition, the increasing richness of material life makes consumers no longer focus too much on meeting the lowest level needs when purchasing products or services. Instead, they are eager to show their unique identity and taste by purchasing products with personalized colors when products with eco-labels not only meet their commercial value but are also an extension of their status or their own personality [28,29,66].

#### 4.2.2. Influence of Green Product Attributes and Marketing Strategy

In addition to the objective reasons (e.g., socio-demographics) and the subjective reasons (e.g., psychological activities, consumption habits, lifestyle), the attributes of products are also the focus of consumers’ purchase intentions. The eco-label that highlights the sustainability of products is the most important factor affecting consumers’ purchase of products, so the product price premium does not have a significant moderating effect on the relationship between the eco-label and purchase intention [12]. Degirmenci and Breitner [14] also found that compared with the price and mileage, the environmental attributes of electric vehicles could better improve consumers’ purchase intention.

Consumers’ preferences for different product attributes are also significantly different according to the various performance of each product. For example, consumers are more concerned about the reliability of the source and the safety of production when buying green food [26,122]. For sustainable clothing, consumers do not only pay attention to the quality, but they also pay more attention to the unique personality of the product when displaying the clothing to others [29,30]. As for electric vehicles, on the one hand, the price and performance are the focus of consumers’ attention [14]; on the other hand, the personality and status of vehicles also have a significant impact on consumers’ choice [24,28]. The health attributes and cost performance are the most widely considered factors when consumers buy household durable goods, such as green housing, green furniture, and energy-saving household appliances [13,31,35]. The country of origin is also one of the consumer considerations when they buy green skincare products [39].

The public attribute of green purchase behavior inevitably impacts consumers’ enthusiasm for green products. Various marketing strategies, such as eco-labels and advertisements, have been applied by businesses to avoid poor sales of green products. Green packaging and an eco-label are the most intuitive means to display product attributes, as these directly provide information, such as the production cycle, origin, and environmental footprint, in which consumers are more interested. The more consumers know about the product, the more willing they are to buy it [80,123]. Moreover, compared with other shapes of brand labels, circular eco-labels are more likely to stimulate consumers’ purchase desire [84]. Advertising is another marketing method with stronger guidance and a wider promotion scope. The advertising appeals of green products are subdivided into abstract appeal (describing product features in a more general or subjective way) and concrete appeal (describing product attributes in a more detailed and objective way). The publicity effect of the abstract appeal is better than that of concrete appeal when the attributes of green products are related to the interests of consumers or others [85]. Therefore, businesses should focus on disseminating the environmental protection value of green products to the public and highlighting their design concept to show their personality and value in a different way to traditional products.

However, businesses should seek truth from the facts and put an end to excessive publicity and false publicity in their marketing. In recent years, many enterprises have tried to repair their public reputation or further enhance it by creating an image of environmental protection. “Greenwashing” incidents have occurred frequently, which is a serious blow to the enthusiasm of consumers for buying green products. Especially for people who are highly concerned about environmental problems, the negative impact of “greenwashing” is more significant [56]. The establishment of trust of product brand in the public mind may take years or even decades of word-of-mouth publicity and quality accumulation, but the destruction of trust usually takes only one incident of enterprise fraud. Especially for food products, the message credibility of advertising plays a more significant role in improving consumers’ payment intention than product quality and product sustainability [86].

#### 4.2.3. Influence of Social Factors

Consumers’ behavior inevitably has to involve contact with the people around them, which determines the social attributes of people. Economists put forward the concept of the “economic man”, which asserts that people will make rational behavior decisions after weighing the pros and cons, but the attributes of the “social man” determine that people are doomed to be influenced by their social environment and the groups around them when making behavior decisions, and the most profound influence is social culture. Generally speaking, consumers who emphasize collectivism are more likely to buy sustainable products than those who emphasize individualism. In particular, consumers who value vertical collectivism think their environmental behavior plays a leading role in social groups, so they are more willing to adopt green consumption to set a positive example. Halder et al. [73] took several European countries (Finland, Germany, Portugal, and America) as their research objects and found that the consumers with a collective and long-term development orientation have a higher green behavior tendency than those who value individualism and a short-term development orientation. The study on the green purchase intention of Indian residents by Fleith and Duarte [124] also supported this finding. In contrast to the residents focusing on their own benefits in Western countries, Chinese people emphasize their own group goals, so they will do what others expect them to do in order to integrate into the social group [3].

Social capital, in contrast to other forms of capital, is rooted in the framework of interpersonal relationships and is usually used purposefully in action [125]. Social media publicity often plays a guiding role in consumers’ attitude towards environmental protection products. Especially for food products, the good reputation established by mass communication is the key factor influencing consumers’ purchase intention of environmental protection food [4,55]. Taking the marketing strategy of the gourmet Meituan app as an example, the comments of the consumers who have purchased their goods are their best advertisement; they have a high degree of credibility for the potential consumers of certain kinds of products.

Social capital is subdivided into bonding capital and bridging capital. Although bonding capital (small and closely related groups, such as a family) is still significant in personal daily life, bridging capital (groups with common interests but not so closely connected) is purposefully used and has a great impact on people’s daily life under the background of network development. Taking YouTube, a popular social software, as an example, many celebrities share their daily life and write comments on the social platform to form a parasocial interaction with their audience. Some beauty bloggers will share their makeup or dressing skills and recommend some products to the public on YouTube. Users following or subscribing to some famous bloggers’ YouTube accounts trust those bloggers and are more likely to buy the products they recommend. Clothing is one of the most easily followed products, and the impact of social capital on these products cannot be underestimated. Kim and Kang [91] investigated the purchase intention of Korean YouTube users for sustainable fashion products. The results indicate that parasocial interaction has a significant impact on bonding and bridging. Bonding has no significant impact on sustainable products, but the effect of bridging is of great significance. This significance is apparent not only in YouTube but also in Weibo, which has more than 400 million active accounts, and in many other apps such as Douyin and Xiaohongshu. More and more social software apps have become important reference platforms for consumers when they shop. This is especially true for green products that are relatively new products; the recommendations of social media celebrities or hot comments are undoubtedly the strongest shopping motivators for consumers who have never had a purchase experience of that product. Therefore, scholars should not concentrate their social capital on communities and other small groups closely related to consumers, they should bring all kinds of new media with value and aesthetic guidance into the research scope when studying green consumption behavior so they are able to explore consumers’ deeper consumption desires.

## 5. Conclusions

We have systematically reviewed the empirical papers on green purchase behavior from 2015 to 2020 and summarized and analyzed the literature from multiple perspectives. The following main conclusions were drawn:

(1) Most of these papers assume that consumers are rational and social people and that green purchase is an activity decision made by consumers through a theoretical mechanism or a fragmented environment.
(2) The research on green purchase behavior in specific countries has mainly concentrated on China, India, Australia, and Europe, but there are few papers focusing on green purchase in a cross-cultural context.
(3) There are many papers on the purchase behavior of those buying general green products. Although clothing, food, vehicles, and housing are all involved, there is obviously more research on food than other types.
(4) The applicability of consumer theory to the study of green purchase behavior is relatively high, and some scholars have expanded and revised the theory by focusing on the psychological factors, as well as the combination of internal and external factors.
(5) The factors influencing green consumers could be divided into three dimensions: individual factors, product attributes and marketing strategy, and social factors, among which the relevant research on individual factors is dominant.

## 6. Limitations and Future Research Directions

Our research also has some limitations. First, the papers we selected were mainly from the Scopus database, which cannot cover all the relevant literature. Scholars can synthesize the research data from multiple databases when conducting a literature review in the future. Second, we used a keyword search to select the matters of interest to us from the literature, which were green product, green purchase, green buying, sustainable products, sustainable consumption, sustainable purchase, and willingness to pay. Future researchers can add keywords such as green attributes, organic food, and sustainable clothing. Third, the papers we analyzed were mainly from 2015 to 2020, and researchers could extend the research time range in the future. Fourth, our research regarded all green product purchase behavior as a category, analyzed all the theories and influencing factors, and did not specifically analyze green products of certain types such as food or clothing. We did not systematically review the literature on green product purchase behavior from a single perspective such as attributes or personal factors.

According to the analysis and summary of the relevant papers, we find that there are some key research directions for the future, and these are set out below.

### 6.1. Research Object

Most of the research on consumers’ green purchase behavior adopted a questionnaire survey where “green product purchase intention” and “willingness to pay” were taken as the final explanatory variables. However, consumers may enhance their environmental protection awareness out of “vanity” or “peace of mind”, overestimate their awareness and sense of responsibility for environmental protection, and mistakenly think they are so environmentally conscious that they are willing to sacrifice their own interests to buy sustainable products, which makes the accuracy of the questionnaire data debatable. When collecting the questionnaire data, scholars can choose those consumers who have already purchased sustainable products as the research objects, which can weaken the subjectivity of consumers in answering the questionnaire to a certain extent.

### 6.2. Dynamic Research

The scholars’ research data on green purchase behavior are cross-sectional data, whether they adopt a field experiment or a questionnaire survey, which can only reflect the psychological activities of consumers at a certain moment; the impact of external environmental changes on consumers, such as education and policy, are ignored. Especially in today’s society with the rapid development of networks, consumers’ consumption preference for certain products changes rapidly, so it is difficult for the research methodology of questionnaire or experiment to meet the actual research needs for horizontal data. Scholars can continuously track consumers’ purchase behavior and vertically study the impact of changes in the external environment on consumers’ green product purchase behavior in the future.

### 6.3. Cross-Cultural Research

At present, the number of research papers in the context of developing and developed countries is about equal, but there is less related research in a cross-cultural context. There are obvious differences in the economic conditions, culture, and traditional customs between developed countries, represented mainly by Europe and the United States, and developing countries, such as China and India. Meaningful references for marketers to develop differentiated marketing strategies could be obtained by comparing the research on the green purchase behavior of consumers under different living environments and national systems.

### 6.4. Multiple-Products Research

Although food is the most daily used product of human beings, it is only one part of the realization of sustainable development. Scholars will focus on housing, furniture, home appliances, travel, and other products and services that cover all aspects of life in the future.

### 6.5. Research on Crossing the Fixed Paradigm

The research paradigm of consumers’ green purchase behavior mainly focuses on the behavior decisions of consumers that follow the analysis of the relevant literatures, which assumes that consumers are rational people and make decisions to maximize their benefits after weighing up various factors or under the constraints of values or individual norms. Therefore, TPB, VAB, and VBN have been the most widely used consumer theories. However, the use of these models puts consumers in a vacuum and pays too much attention to the psychological factors of consumers while ignoring the influence of external factors. ABC, which takes external factors into the research scope, has been widely used in order to overcome the limitations of the abovementioned theory. However, the factors and paths for influencing consumers’ green purchase behavior are more complex, so using TPB, VAB or other theoretical models alone to explain consumers’ purchase behavior is limited. Therefore, researchers could integrate multiple theories to explain consumers’ purchase decisions in respect of green products in the future.

In addition, most scholars generally think that the decision-making process of purchase behavior is the result of the linear effect of various factors when exploring the green purchase behavior of consumers from a certain perspective, such as values, consumer innovation, eco-label, social capital, etc., or put consumers in a certain social background. Therefore, regression analysis, path analysis, and structural equation model are more commonly used empirical means in their research process. However, consumers’ purchase behavior is affected by various factors such as psychological and external environment, which makes their decision-making process more complicated. As a result, the simple linear causality model may have limited interpretation in explaining their shopping options, so future researchers could try to use non-linear models to explain consumers’ behavior, which would further understand green purchase behavior.

## Figures and Tables

**Table 1:** Classification and summary according to product category.

Product Category	Region	Consumer Theory	Key Words
**Food**	China; Italy; Germany; Malaysia; Australia; Canada; European Union; Korea; Romania	TPB; VAB; TPB; ABC	Face consciousness, Group conformity, Trust; Benefit type, Product quality, Green self-identity, Pollution, Food neophobia, Food technology neophobia, Origin, Health orientation, Sensory appeal, Value, Satisfaction, Perceived power, Perceived wealth, Past behavior, Price premium, Eco-label, Availability
**Vehicles**	Brazil; Germany; China; America; Japan	VAB; TRA	Attributes of vehicles, Environment performance, Range confidence, Price, Self-expressive benefits, Perceived value, Climate and geographical features, Social network, Policy incentives, Pollution identity, Status motivation, Cultural values,
**Clothing**	Germany; Korea; Italy; America; Britain; India;	VAB; VBN; TRA	Values, Fashion consciousness, Price sensitivity, Social norm, Fashion brand, Beliefs, Social media, Peer influence, Self-expressiveness
**Housing**	Australia; China	TPB	Green consumer identity, Anchoring price, Social norm, Values, Risk, Socio-demographics, Public information
**Furniture**	China; Brazil	TPB	Environmental consciousness, Physical health concern, Past experience, Perceived value, Price elasticity, Attributes of products, Socio-demographics
**Household Appliance**	Malaysia	TPB	Environmental concern; Environmental knowledge; Moral norm, Socio-demographics, Trust in environment
**Others (Skincare, Bioplastic products, Green energy)**	China; America; India	TPB	Country of origin, Price sensitivity, Green self-identity, Self-congruity, Green perceived value

**Table 2:** Consumer theory with high application frequency.

Theory	Authors
TRA	Policarpo and Aguiar [28]; Zhao et al. [32]
TPB	Xu et al. [7]; Patel et al. [8]; Xu et al. [9]; Carfora et al. [26]; Setyawan et al. [33]; Choi and Johnson [34]; Sreen et al. [35]; Qi and Ploeger [36]; Liobikiene et al. [37]; Yadav and Govind [38]; Hsu et al. [39]; Hojjat and Behnaz [40]; Madeline et al. [41]; Paul et al. [42]; Sultan et al. [43]; Johe and Bhullar [44]; Sharma and Foropon [45]
VAB	Cheung and To [6]; Trivedi et al. [10]; Degirmenci and Breitner [14]; Kathleen et al. [30]; Shin et al. [46]; Tan et al. [47]; Lee et al. [48]; Woo and Kim [49]
NAM	Yu et al. [50]
VBN	Han et al. [51]; Kim et al. [52]
ABC	Silvia and Maria [5]; Chekima et al. [12]; Myriam et al. [53]; Megavannan et al. [54]; Sergio et al. [55]; Zhang et al. [56]

**Table 3:** Key factors influencing green purchase behavior.

Drivers	Variables	Authors
**Individual Factors**		
Psychological factors: Attitude, Awareness, Beliefs, Values, Norm, Perception	Attitude toward environment/green products, Awareness of green products/environment, Confidence, Eco-literacy, Emotions, Environmental concern/consciousness/ethics/responsibility, Perceived behavior control, Perceived consumer effectiveness, Subjective/Moral norm, Willingness to pay premium, Expectation, Health consciousness, Self-identity, Self-image	Mecikalski and Carey [4]; Silvia and Maria [5]; Cheung and To [6]; Patel et al. [8]; Chekima et al. [12]; Silva et al. [23]; Carfora et al. [26]; Hojjat and Behnaz [40]; Shin et al. [46]; Tan et al. [47]; Megavannan et al. [54]; Arminda et al. [57]; Shiel et al. [58]; Shao and Unal [59]; Wei et al. [60]; Lai and Cheng [61]; Sangroya and Navak [62]; Yatish and Zillur [63]; Tong et al. [64]; Laureti and Benedetti [65]; Wei and Jung [66]; Ciasullo et al. [67]; Khare and Sadachar [68]; Osburg et al. [69]; Confente et al. [70]
Habits, Experiences, Lifestyle	Face/Status consciousness, Green involvement, Interpersonal differentiation, Past purchase experiences, Knowledge of environment, Health status, Healthy life habits	Jung et al. [3]; Wang et al. [11]; Silva et al. [23]; Koklic et al. [27]; Park and Lin [29]; Qi and Ploeger [36]; Laureti and Benedetti [65]; Wei and Jung [66]; Khare and Sadachar [68]; Osburg et al. [69]; Khare [71]
Socio-demographics	Education Level, Age, Gender, Occupation, Family structure	Silvia and Maria [5]; Sreen et al. [35]; Shiel et al. [58]; Pícha and Navrátil [72]; Halder et al. [73]; Shahsavar et al. [74]; Wang et al. [75]
**Product Attributes and Marketing**		
Product attribute: Availability, Product quality, Packaging, Origin	Availability, Energy and Material, Packaging, Product attributes, Perceived risks/trust, Product price, Origin, Impact on society	Silvia and Maria [5]; Carfora et al. [26]; Kathleen et al. [30]; Hsu et al. [39]; Hojjat and Behnaz [40]; Sultan et al. [43]; Megavannan et al. [54]; Shao and Unal [59]; Chekima et al. [76]; Matsumoto et al. [77]; Lee et al. [78]; See and Balaji [79]; Hao et al. [80]; William et al. [81];
Marketing: Eco-label, Message credibility, Promotion, Sales channels	Eco-label, Message credibility, Advertisement, Green certification, Mass media, Marketing influence, Green word-to-mouth	Wang et al. [11]; Zhang et al. [56]; Lai and Cheng [61]; Yatish and Zillur [63]; Cai et al. [82]; Zhou et al. [83]; Xu et al. [84]; Yang et al. [85]; Jäger and Anja [86]; Yatish and Zillur [87]
**Social Influence**		
Social norm	Social norm, Peer Influence, Culture, Organization	Wang et al. [11]; Afzaal et al. [24]; Sreen et al. [35]; Qi and Ploeger [36]; Khare [71]; Halder et al. [73]; Yatish and Zillur [87]; Liobikiene et al. [88]; Lu et al. [89]
Social capital	Social capital, Media, Place identity	Sidharth et al. [22]; Zhao et al. [32]; Lee et al. [48]; Sergio et al. [55]; Zhang et al. [90]; Kim and Kang [91]; Yang and Zhang [92]

**Figure A1:**
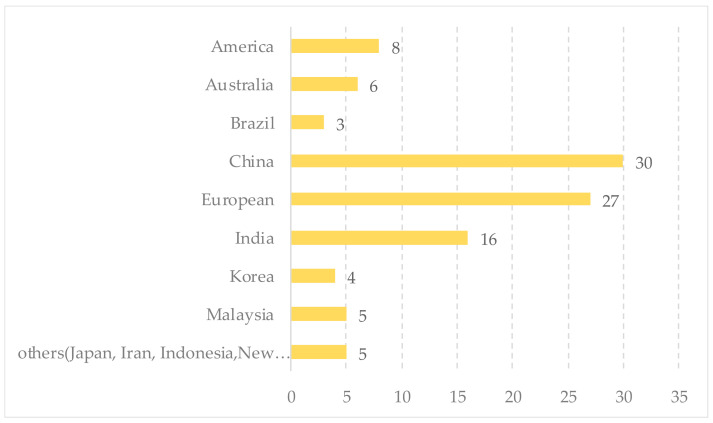
Distribution of papers across region.

## Appendix A

**Table A1:** Summary of literature search results and number of publications.

Journal	Period						Total
	2015	2016	2017	2018	2019	2020	
Appetite					2		2
Applied Energy				1			1
Asia Pacific Journal of Marketing and Logistics				1			1
Business Strategy and the Environment				1			1
British Food Journal					1		1
Clothing and Textiles Research Journal		1					1
Ecological Economics		3	1		1		5
Energy Policy			1				1
Food Quality and Preference					1	1	2
Food Research International					1		1
Forest Policy and Economics			1				1
Global Ecology and Conservation					1		1
International Journal of Consumer Studies	1		1				2
International Journal of Recent Technology and Engineering					1		1
Journal of Business Ethics	1						1
Journal of Business Research	1	2		2	2	1	8
Journal of Cleaner Production		5	6	7	7	7	32
Journal of Consumer Marketing	1						1
Journal of Hospitality and Tourism Management			1				1
Journal of International Consumer Marketing		1					1
Journal of Retailing and Consumer Services		1	1	3	1	1	7
Journal of Travel and Tourism Marketing			1				1
Management					2		2
Marketing Intelligence and Planning	1						1
Procedia Economics and Finance		1					1
Procedia Social and Behavioral Sciences	1						1
Problems and Perspectives in Management				1			1
Resources, Conservation & Recycling					1	2	3
Science of the Total Environment				1			1
Sustainability			2	1		3	6
Sustainable Cities and Society			1				1
Sustainable Development		1			1		2
Sustainable Production and Consumption			1		1		2
The Social Science Journal		1					1
Transport Policy			1				1
Transportation Research			1				1
**Total**	6	16	19	18	23	15	97

## References

[1] Sheng G.H., Ge W.D., Tang L. (2018). The impact of consumer’s environmental responsibility on green product purchase behavior—A case of energy saving household appliances. Forum Stat. Inf..

[2] Groening C., Sarkis J., Zhu Q. (2018). Green marketing consumer-level theory review: A compendium of applied theories and further research directions. J. Clean. Prod..

[3] Jung H.J., Choi Y.J., Oh K.W. (2020). Influencing Factors of Chinese Consumers’ Purchase Intention to Sustainable Apparel Products: Exploring Consumer ‘Attitude–Behavioral Intention’ Gap. Sustainability.

[4] Mecikalski R.M., Carey L.D. (2018). Exploring the Consumer Behavior of Intention to Purchase Green Products in Belt and Road Countries: An Empirical Analysis. Sustainability.

[5] Silvia C., Maria A.P. (2020). Sustainable consumption in the circular economy. An analysis of consumers’ purchase intentions for waste-to-value food. J. Clean. Prod..

[6] Cheung M.F.Y., To W.M. (2019). An extended model of value-attitude-behavior to explain Chinese consumers’ green purchase behavior. J. Retail. Consum. Serv..

[7] Xu X., Hua Y., Wang S., Xu G. (2020). Determinants of consumer’s intention to purchase authentic green furniture. Resour. Conserv. Recycl..

[8] Patel J.D., Rohit R.H., Yagnik A. (2020). Self-identity and internal environmental locus of control: Comparing their influences on green purchase intentions in high-context versus low-context cultures. J. Retail. Consum. Serv..

[9] Xu X., Wang S., Yu Y. (2018). Consumer’s intention to purchase green furniture: Do health consciousness and environmental awareness matter?. Sci. Total Environ..

[10] Trivedi R.H., Patel J.D., Acharya N. (2018). Causality analysis of media influence on environmental attitude, intention and behaviors leading to green purchasing. J. Clean. Prod..

[11] Wang J., Bao J., Wang C., Wu L. (2017). The impact of different emotional appeals on the purchase intention for green products: The moderating effects of green involvement and Confucian cultures. Sustain. Cities Soc..

[12] Chekima B.C., Syed Khalid Wafa S.A.W., Igau O.A., Chekima S., Sondoh S.L. (2016). Examining green consumerism motivational drivers: Does premium price and demographics matter to green purchasing?. J. Clean. Prod..

[13] Li Q., Long R., Chen H. (2018). Differences and influencing factors for Chinese urban resident willingness to pay for green housings: Evidence from five first_tier cities in China. Appl. Energy.

[14] Degirmenci K., Breitner M.H. (2017). Consumer purchase intentions for electric vehicles: Is green more important than price and range?. Transp. Res..

[15] Ebru T.K., Inci D., Alev K.A. Green purchase intention of young Turkish consumers: Effects of consumer’s guilt, self-monitoring and perceived consumer effectiveness. Proceedings of the 11th International Strategic Management Conference.

[16] Dong X., Liu S., Li H., Yang Z., Liang S., Deng N. (2020). Love of nature as a mediator between connectedness to nature and sustainable consumption behavior. J. Clean. Prod..

[17] Sheng G.H., Yue B.B., Xie F. (2019). Research on driving mechanism of green consumption behavior of Chinese residents from the perspective of environmental co-governance. Forum Stat. Inf..

[18] Sheng G.H., Ge W.D. (2019). A study on the social mechanism driving consumers’ green purchase from the perspective of social interaction. J. Huazhong Agric. Univ..

[19] Bangsa A.B., Schlegelmilch B.B. (2020). Linking sustainable product attributes and consumer decision-making: Insights from a systematic review. J. Clean. Prod..

[20] Yatish J., Zillur R. (2015). Factors Affecting Green Purchase Behaviour and Future Research Directions. Int. Strateg. Manag. Rev..

[21] Liobikienė G., Bernatonienė J. (2017). Why determinants of green purchase cannot be treated equally? The case of green cosmetics: Literature review. J. Clean. Prod..

[22] Sidharth M., Francisco R.-G., Xue F. (2016). Understanding the green buying behavior of younger Millennials from India and the United States: A structural equation modeling approach. J. Int. Consum. Mark..

[23] Silva M.D., Wang P., Kuah A.T.H. (2020). Why Wouldn’t Green Appeal Drive Purchase Intention? Moderation Effects of Consumption Values in the UK and China. J. Bus. Res..

[24] Afzaal A., Guo X., Adnan A., Mehkar S., Muhammad M.F. (2019). Customer motivations for sustainable consumption: Investigating the drivers of purchase behavior for a green-luxury car. Bus. Strategy Environ..

[25] Bin S., Dowlatabadi H. (2005). Consumer lifestyle approach to US energy use and the related CO_2_ emissions. Energy Policy.

[26] Carfora V., Cavallo C., Caso D., Del G.T. (2019). Explaining consumer purchase behavior for organic milk: Including trust and green self-identity within the theory of planned behavior. Food Qual. Prefer..

[27] Koklic M.K., Golob U., Podnar K., Zabkar V. (2019). The interplay of past consumption, attitudes and personal norms in organic food buying. Appetite.

[28] Policarpo M.C., Aguiar E. (2020). How self-expressive benefits relate to buying a hybrid car as a green product. J. Clean. Prod..

[29] Park H.J., Lin L.M. (2020). Exploring attitude–behavior gap in sustainable consumption: Comparison of recycled and upcycled fashion products. J. Bus. Res..

[30] Kathleen J., Lars P., Jacob H., Dirk B. (2018). Green thinking but thoughtless buying? An empirical extension of the value-attitude-behaviour hierarchy in sustainable clothing. J. Clean. Prod..

[31] Fleith D.M.J., Duarte R.J.L., Nogueira C.M. (2016). Influence of perceived value on purchasing decisions of green products in Brazil. J. Clean. Prod..

[32] Zhao L., Lee S.H., Copeland L.R. (2018). Social media and Chinese consumers’ environmentally sustainable apparel purchase intentions. Asia Pac. J. Mark. Logist..

[33] Setyawan A., Noermijati N., Sunaryo S. (2018). Green product buying intentions among young consumers: Extending the application of theory of planned behavior. J. Prob. Perspect. Manag..

[34] Choi D., Johnson K.K.P. (2019). Influences of environmental and hedonic motivations on intention to purchase green products: An extension of the theory of planned behavior. Sustain. Prod. Consum..

[35] Sreen N., Purbey S., Sadarangani P. (2018). Impact of culture, behavior and gender on green purchase intention. J. Retail. Consum. Serv..

[36] Qi X., Ploeger A. (2019). Explaining consumers’ intentions towards purchasing green food in Qingdao, China: The amendment and extension of the theory of planned behavior. Appetite.

[37] Liobikiene G., Mandravickaite J., Bernatoniene J. (2016). Theory of planned behavior approach to understand the green purchasing behavior in the EU: A cross-cultural study. Ecol. Econ..

[38] Yadav R., Pathak G.S. (2016). Young consumers’ intention towards buying green products in a developing nation: Extending the theory of planned behavior. J. Clean. Prod..

[39] Hsu C.-L., Chang C.-Y., Yansritakul C. (2017). Exploring purchase intention of green skincare products using the theory of planned behavior: Testing the moderating effects of country of origin and price sensitivity. J. Retail. Consum. Serv..

[40] Hojjat M., Behnaz K. (2016). Investigating the Factors Affecting Female Consumers’ Willingness toward Green Purchase Based on the Model of Planned Behavior. Procedia Econ. Financ..

[41] Madeline J., Georgia W.-M., Angela P. (2019). Using the theory of planned behaviour to predict intentions to purchase sustainable housing. J. Clean. Prod..

[42] Paul J., Modi A., Patel J. (2016). Predicting green product consumption using theory of planned behavior and reasoned action. J. Retail. Consum. Serv..

[43] Sultan P., Tarafder T., Pearson D., Henryks J. (2020). Intention-behaviour gap and perceived behavioural control-behaviour gap in theory of planned behaviour: Moderating roles of communication, satisfaction and trust in organic food consumption. Food Qual. Prefer..

[44] Johe M., Bhullar N. (2016). To buy or not to buy: The roles of self-identity, attitudes, perceived behavioral control and norms in organic consumerism. Ecol. Econ..

[45] Sharma A., Foropon C. (2019). Green product attributes and green purchase behavior: A theory of planned behavior perspective with implications for circular economy. Management.

[46] Shin Y.H., Moon H., Jung S.E., Severt K. (2017). The effect of environmental values and attitudes on consumer willingness to pay more for organic menus: A value-attitude-behavior approach. J. Hosp. Tour. Manag..

[47] Tan C.-S., Ooi H.-Y., Goh Y.-N. (2017). A moral extension of the theory of planned behavior to predict consumers’ purchase intention for energy-efficient household appliances in Malaysia. Energy Policy.

[48] Lee C.K.C., Levy D.S., Ya C.S.F. (2015). How does the theory of consumption values contribute to place identity and sustainable consumption?. Int. J. Consum. Stud..

[49] Woo E., Kim Y.G. (2019). Consumer attitudes and buying behavior for green food products. Br. Food J..

[50] Yu T.-Y., Yu T.-K., Chao C.-M. (2017). Understanding Taiwanese undergraduate students’ pro-environmental behavioral intention towards green products in the fight against climate change. J. Clean. Prod..

[51] Han H., Hwang J., Lee M.J. (2017). The value–belief–emotion–norm model: Investigating customers’ eco-friendly behavior. J. Travel. Tour. Mark..

[52] Kim H.J., Kim J.Y., Oh K.W., Jung H.J. (2016). Adoption of Eco-Friendly Faux Leather: Examining Consumer Attitude with the Value–Belief–Norm Framework. Cloth. Text. Res. J..

[53] Myriam E., Fahri K., Emine S. (2016). Exploring pro-environmental behaviors of consumers: An analysis of contextual factors, attitude, and behaviors. J. Bus. Res..

[54] Megavannan R., Arivazhagan R., Name G. (2019). A study on green products buying decision among chennai people with respect to their ecological consciousness and challenges to buy. Int. J. Recent Technol. Eng. (IJRTE).

[55] Sergio V., Federica B., Cristina G., Gioacchino B., Charles O.R., Ignazio P., Mario S., Vito P. (2020). Consumers’ Perception and Willingness to Pay for Eco-Labeled Seafood in Italian Hypermarkets. Sustainability.

[56] Zhang L., Li D., Cao C., Huang S. (2018). The influence of greenwashing perception on green purchasing intentions: The mediating role of green word-of-mouth and moderating role of green concern. J. Clean. Prod..

[57] Arminda D.P., Chris S., Helena A. (2019). A new model for testing green consumer behaviour. J. Clean. Prod..

[58] Shiel C., Paço A.D., Alves H. (2020). Generativity, sustainable development and green consumer behaviour. J. Clean. Prod..

[59] Shao J., Ünal E. (2019). What do consumers value more in green purchasing? Assessing the sustainability practices from demand side of business. J. Clean. Prod..

[60] Wei S., Ang T., Jancenelle V.E. (2018). Willingness to pay more for green products: The interplay of consumer characteristics and customer participation. J. Retail. Consum. Ser..

[61] Lai C.K.M., Cheng E.W.L. (2016). Green purchase behavior of undergraduate students in Hong Kong. Soc. Sci. J..

[62] Sangroya D., Nayak J.K. (2017). Factors influencing buying behaviour of green energy consumer. J. Clean. Prod..

[63] Yatish J., Zillur R. (2019). Consumers’ Sustainable Purchase Behaviour: Modeling the Impact of Psychological Factors. Ecol. Econ..

[64] Tong Q., Anders S., Zhang J., Zhang L. (2020). The roles of pollution concerns and environmental knowledge in making green food choices: Evidence from Chinese consumers. Food Res. Int..

[65] Laureti T., Benedetti I. (2018). Exploring pro-environmental food purchasing behaviour: An empirical analysis of Italian consumers. J. Clean. Prod..

[66] Wei X., Jung S. (2017). Understanding Chinese Consumers’ Intention to Purchase Sustainable Fashion Products: The Moderating Role of Face-Saving Orientation. Sustainability.

[67] Ciasullo M.V., Maione G., Torre C., Troisi O. (2017). What about Sustainability? An Empirical Analysis of Consumers’ Purchasing Behavior in Fashion Context. Sustainability.

[68] Khare A., Sadachar A. (2017). Green apparel buying behaviour: A study on Indian youth. Int. J. Consum. Stud..

[69] Osburg V.-S., Yoganathan V., Brueckner S., Toporowski W. (2020). How detailed product information strengthens eco-friendly consumption. Manag. Decis..

[70] Confente I., Scarpi D., Russo I. (2020). Marketing a new generation of bio-plastics products for a circular economy: The role of green self-identity, self-congruity, and perceived value. J. Bus. Res..

[71] Khare A. (2015). Antecedents to green buying behaviour: A study on consumers in an emerging economy. Mark. Intell. Plan..

[72] Pícha K., Navrátil J. (2019). The factors of Lifestyle of Health and Sustainability influencing pro-environmental buying behaviour. J. Clean. Prod..

[73] Halder P., Hansen E.N., Kangas J., Laukkanen T. (2020). How national culture and ethics matter in consumers’ green consumption values. J. Clean. Prod..

[74] Shahsavar T., Kubeš V., Baran D. (2020). Willingness to pay for eco-friendly furniture based on demographic factors. J. Clean. Prod..

[75] Wang X., Li W., Song J., Duan H., Fang K., Diao W. (2020). Urban consumers’ willingness to pay for higher-level energy-saving appliances: Focusing on a less developed region. Resour. Conserv. Recycl..

[76] Chekima B., Oswald A.I., Wafa S.A.W.S.K., Chekima K. (2017). Narrowing the gap: Factors driving organic food consumption. J. Clean. Prod..

[77] Matsumoto M., Chinen K., Endo H. (2018). Remanufactured auto parts market in Japan: Historical review and factors affecting green purchasing behavior. J. Clean. Prod..

[78] Lee E.J., Bae J., Kim K.H. (2019). The effect of environmental cues on the purchase intention of sustainable products. J. Bus. Res..

[79] See K.G., Balaji M.S. (2016). Linking green skepticism to green purchase behavior. J. Clean. Prod..

[80] Hao Y., Hao L., Chen H., Sha Y., Ji H., Fan J. (2019). What affect consumers’ willingness to pay for green packaging? Evidence from China. Resour. Conserv. Recycl..

[81] William R., Ourania T., Louise M. (2019). Sustainability cues on packaging: The influence of recognition on purchasing behavior. J. Clean. Prod..

[82] Cai Z., Xie Y., Aguilar F. (2017). Eco-label credibility and retailer effects on green product purchasing intentions. For. Policy Econ..

[83] Zhou S., Wang H., Li S., Chen Y., Wu J. (2019). Carbon labels and “horizontal location effect”: Can carbon labels increase the choice of green product?. J. Glob. Ecol. Conserv..

[84] Xu L., Yu F., Ding X. (2020). Circular-Looking Makes Green-Buying: How Brand Logo Shapes Influence Green Consumption. Sustainability.

[85] Yang D., Lu Y., Zhu W., Su C. (2015). Going green: How different advertising appeals impact green consumption behavior. J. Bus. Res..

[86] Jäger A.-K., Anja W. (2020). Can you believe it? The effects of benefit type versus construal level on advertisement credibility and purchase intention for organic food. J. Clean. Prod..

[87] Yatish J., Zillur R. (2017). Investigating the determinants of consumers’ sustainable purchase behaviour. Sustain. Prod. Consum..

[88] Liobikiene G., Grincevičienė S., Bernatoniene J. (2017). Environmentally friendly behaviour and green purchase in Austria and Lithuania. J. Clean. Prod..

[89] Lu L.C., Chang H.-H., Chang A. (2015). Consumer Personality and Green Buying Intention: The Mediate Role of Consumer Ethical Beliefs. J. Bus. Ethics.

[90] Zhang L., Sun C., Liu H., Zheng S. (2016). The role of public information in increasing homebuyers’ willingness-to-pay for green housing: Evidence from Beijing. J. Ecol. Econ..

[91] Kim J., Kang S. (2020). How social capital impacts the purchase intention of sustainable fashion products. J. Bus. Res..

[92] Yang X., Zhang L. (2020). Diagnose barriers to sustainable development: A study on “desensitization” in urban residents’ green purchasing behavior. Sustain. Dev..

[93] Fishbein M., Ajzen I. (1977). Belief, Attitude, Intention and Behavior: An Introduction to Theory and Research.

[94] Ajzen I. (1991). The theory of planned behavior. Organ. Behav. Hum. Dec..

[95] Mathieson K. (1991). Predicting user intentions: Comparing the technology acceptance model with the theory of planned behavior. Inf. Syst. Res..

[96] Moser A.K. (2015). Thinking green, buying green? Drivers of pro-environmental purchasing behavior. J. Consum. Mark..

[97] Rezai G., Teng P.K., Mohamed Z., Shamsudin M.N. (2012). Consumers’ Awareness and Consumption Intention towards Green Foods. Afr. J. Bus. Manag..

[98] Yadav R., Pathak G.S. (2017). Determinants of Consumers’ Green Purchase Behavior in a Developing Nation: Applying and Extending the Theory of Planned Behavior. Ecol. Econ..

[99] Lao K. (2013). Research on the influence mechanism of consumer innovation on green consumption behavior. Nankai Manag. Rev..

[100] Pradeep K., Justin P., Rajesh S. (2019). The moderating influence of environmental consciousness and recycling intentions on green purchase behavior. J. Clean. Prod..

[101] Schwartz S.H., Berkowitz L. (1977). Normative Influences on Altruism. Advances in Experimental Social Psychology.

[102] Sheth J.N., Newman B.I., Gross B.L. (1991). Why we buy what we buy: A theory of consumption values. J. Bus. Res..

[103] Gonçalves H.M., Lourenço T.F., Silva G.M. (2016). Green buying behavior and the theory of consumption values: A fuzzy-set approach. J. Bus. Res..

[104] Mohd S.N. (2016). Consumer environmental concern and green product purchase in Malaysia: Structural effects of consumption values. J. Clean. Prod..

[105] Awuni J.A., Du J. (2016). Sustainable Consumption in Chinese Cities: Green Purchasing Intentions of Young Adults Based on the Theory of Consumption Values. Sustain. Dev..

[106] Stern P.C. (2000). Toward a Coherent Theory of Environmentally Significant Behavior. J. Soc. Issues.

[107] Dietz T., Stern P.C., Guagnano G.A. (1997). Social structural and social psychological bases of environmental concern. Environ. Behav..

[108] Stern P.C., Dietz T., Guagnano G.A. (1995). The new environmental paradigm in social psychological perspective. Environ. Behav..

[109] Kaiser F.G., Hubner G., Bogner F.X. (2005). Contrasting the theory of planned behavior with the value-belief-norm model in explaining conservation behavior. J. Appl. Soc. Psychol..

[110] Andersson L., Shivarajan S., Blau G. (2005). Enacting ecological sustainability in the multinational corporation: A test of an adapted value-belief-norm framework. J. Bus. Ethics.

[111] Bamberg S., Moser C. (2007). Twenty Years after Hines. Hungerford, and Tomera: A New Meta-analysis of Psycho-social Determinants of Pro-environmental Behavior. J. Environ. Psychol..

[112] Klöckner C.A., Blöbaum A. (2010). A comprehensive action determination model: Toward a broader understanding. J. Environ. Psychol..

[113] Stern P.C., Oskamp S., Stkols D., Altman I. (1987). Managing scarce environmental resources. Handbook of Environmental Psychology.

[114] Guagnano G.A., Stern P.C., Dietz T. (1995). Influences on Attitude-Behavior Relationships: A Natural Experiment with Curbside Recycling. Environ. Psychol. Nonverbal. Behav..

[115] Peng Y. (2013). A review of the research on Influencing Factors of environmental behavior at home and abroad. China. Popul. Resourc. Environ..

[116] Brand K.W., Redclifi M., Woodgate G. (1997). Environmental Consciousness and Behaviour: The Greening of Lifestyles. The International Handbook of Environmnental Sociology.

[117] Wang J. (2013). The influence of resource conservation consciousness on resource conservation behavior: An interactive and moderating effect model in the context of Chinese culture. J. Manag. World.

[118] Habich-Sobiegalla S., Kostka G., Anzinger N. (2017). Citizens’ electric vehicle purchase intentions in China: An analysis of micro-level and macro-level factors. Transp. Policy.

[119] Liang D., Hou C., Jo M.-S., Sarigöllü E. (2019). Pollution avoidance and green purchase: The role of moral emotions. J. Clean. Prod..

[120] Jaiswal D., Kant R. (2018). Green purchasing behaviour: A conceptual framework and empirical investigation of Indian consumers. J. Retail. Consum. Serv..

[121] Wang Y., Li Y., Zhang J., Su X. (2019). How impacting factors affect Chinese green purchasing behavior based on Fuzzy Cognitive Maps. J. Clean. Prod..

[122] Polimeni J.M., Iorgulescu R.I., Mihnea A. (2018). Understanding consumer motivations for buying sustainable agricultural products at Romanian farmers markets. J. Clean. Prod..

[123] Cerri J., Testa F., Rizzi F. (2017). The more I care, the less I will listen to you: How information, environmental concern and ethical production influence consumers’ attitudes and the purchasing of sustainable products. J. Clean. Prod..

[124] Fleith d.M.J., Duarte R.J.L. (2017). Environmentally sustainable innovation: Expected attributes in the purchase of green products. J. Clean. Prod..

[125] Lin N. (2001). Social Capital: A Theory of Social Structure and Action.

